# Prognostic role of serum concentrations of high-sensitivity C-reactive protein in patients with metastatic colorectal cancer: results from the ITACa trial

**DOI:** 10.18632/oncotarget.7166

**Published:** 2016-02-03

**Authors:** Andrea Casadei Gardini, Silvia Carloni, Emanuela Scarpi, Paolo Maltoni, Romolo M. Dorizzi, Alessandro Passardi, Giovanni Luca Frassineti, Pietro Cortesi, Maria Benedetta Giannini, Giorgia Marisi, Dino Amadori, Alessandro Lucchesi

**Affiliations:** ^1^ Department of Medical Oncology, Istituto Scientifico Romagnolo per lo Studio e la Cura dei Tumori (IRST) IRCCS, Meldola, Italy; ^2^ Biosciences Laboratory, Istituto Scientifico Romagnolo per lo Studio e la Cura dei Tumori (IRST) IRCCS, Meldola, Italy; ^3^ Unit of Biostatistics and Clinical Trials, Istituto Scientifico Romagnolo per lo Studio e la Cura dei Tumori (IRST) IRCCS, Meldola, Italy; ^4^ Corelab Unit, AVR Laboratory, Cesena, Italy

**Keywords:** metastatic colorectal cancer, high-sensitivity C-reactive protein, thrombosis, progression-free survival, overall survival

## Abstract

Serum levels of C-reactive protein are (CRP) higher in patients with neoplastic conditions and numerous studies have been performed to clarify the etiologic and prognostic role of the high-sensitivity CRP (hs-CRP) in cancer. Our study was conducted on patients enrolled in the prospective randomized “Italian Trial in Advanced Colorectal Cancer (ITACa)” to assess hs-CRP levels and their impact on overall survival (OS) and progression-free survival (PFS). Serum samples from 132 ITACa patients were collected at baseline and 2 months after starting first-line chemotherapy. The supernatant was immediately transferred to cryovials and stored at −80°C. After thawing, hs-CRP was measured with the Cobas c501 analyzer. High levels of hs-CRP (≥ 13.1 mg/L) were associated with poorer median PFS (*p* < 0.0001) and OS (*p* < 0.0001) than low hs-CRP levels (< 13.1 mg/L). hs-CRP values in 107 patients were evaluated again after 2 months of therapy, revealing that patients with low hs-CRP levels in both baseline and second serum samples had the best median PFS and OS. Our study confirms the prognostic value of hs-CRP in patients with metastatic colorectal carcinoma.

## INTRODUCTION

Colorectal cancer (CC) is the third most common cause of cancer death in Western Europe and North America [1]. Interactions between the tumor and the host are increasingly recognized as important determinants of the clinical course of the disease. In particular, the status of the host's immune system has been shown to be of both prognostic and therapeutic relevance in CC and other cancers. Beneficial immune responses and detrimental pro-inflammatory responses have also been reported [2]. A pro-inflammatory immune response to the tumor may cause a systemic inflammatory response in the host which can be detected in untreated patients before surgery by measuring preoperative increases in circulating protein levels such as C-reactive protein (CRP) [3].

CRP, a typical systemic inflammation marker, was first discovered in the plasma of patients during the acute phase of pneumococcal pneumonia [4]. The protein is produced almost exclusively in hepatocytes in response to inflammatory cytokines, such as interleukin (IL)-1, tumor necrosis factor (TNF)-α and, in particular, IL-6 [5, 6]. There is increasing evidence that elevated CRP levels are associated with an increased risk of malignancy [7–10]. Moreover, elevated CRP has been described as a prognostic factor in various tumors, including ovarian and gastroesophageal cancer [11–14].

Some studies have concluded that elevated CRP levels are not an independent negative predictor of survival [15], whereas others have reported that CRP has a prognostic significance in colorectal cancer [16, 17], suggesting the presence of a detrimental systemic inflammatory response. However, these reports were mostly derived from single-center study, analyzing colon and rectal cancer together. [17].

The correlation between CRP levels and prognosis in patients with CRC remains unclear [18, 19]. Several studies, including some conducted in non-neoplastic pathological settings, have found that an increased IL-6 expression is often linked to endothelial impairment, atherosclerosis and coronary artery disease. Whilst levels of CRP seem to be independently associated with a significant risk of cardiovascular disease, their role in predicting venous thromboembolism (VTE) is still controversial.

In CC patients, IL-6 has been described as a key regulator of cancer development, especially in sporadic and inflammation-related tumors [20]. Moreover, in this setting, inflammatory cytokines have been described as a potential cause of endothelial impairment. Interestingly, in experimental models of colitis from inflammatory bowel diseases, one study concluded that IL-6 may induce a hypercoagulable state through enhanced platelet activation [21].

Our study was carried out as a secondary analysis on patients enrolled in the phase III prospective multicenter randomized “Italian Trial in Advanced Colorectal Cancer (ITACa)” (EudraCT no. 2007–004539–44 and registered on ClinicalTrials.gov NCT01878422) [22] to assess high-sensitivity C-reactive protein (hs-CRP) levels at diagnosis and their impact on progression-free (PFS) and overall survival (OS). A further aim was to evaluate the potential role of hs-CRP in predicting a cardiovascular event, in particular, venous thromboembolism (VTE).

## RESULTS

### Patient characteristics

**Figure 1:**
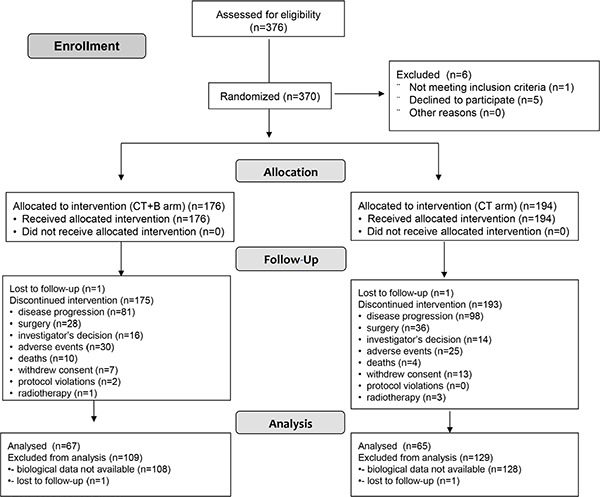
Flow chart of the study

Between 14 November 2007 and 6 March 2012, 132 patients diagnosed with metastatic colorectal cancer were available for baseline hs-CRP analysis (Figure 1): 78 (59.1%) were males and 54 (40.9%) females with a median age at diagnosis of 67 years (range 34–83). Median follow-up was 36 months (range 1–65). The main demographic and clinical characteristics of the studied population are summarized in Table 1. Median PFS was 9.7 (95% CI 9.0–10.9) and median OS was 22.7 months (95% CI 19.3–27.1).

**Table 1:** Patient characteristics (n = 132)

	No. (%)
**Median age**, years (range)	67 (34–83)
**Age**	
< 70	74 (56.1)
≥ 70	58 (43.9)
**Gender**	
Males	78 (59.1)
Females	54 (40.9)
**Performance Status (ECOG)**	
0	110 (83.3)
1–2	22 (16.7)
**Tumor localization**	
Rectum	37 (28.0)
Colon	95 (72.0)
**Stage at diagnosis**	
I–III	31 (23.5)
IV	101 (76.5)
**CT regimen**	
FOLFOX4	79 (59.8)
FOLFIRI	53 (40.2)
**KRAS status**	
Wild type	75 (59.1)
Mutated	52 (40.9)
Unknown/missing	6
**Study treatment**	
CT + B	67 (50.8)
CT	65 (49.2)
**Site of metastases**	
Only liver	37 (28.0)
Other	95 (72.0)
**Comorbidities**	
Cardiovascular	64 (48.1)
Pulmonary	4 (3.0)
Gastrointestinal/hepatobiliary	10 (7.5)
Metabolic/endocrine	25 (18.8)
Musculoskeletal	4 (3.0)
Dermatologic	1 (0.7)
Reproductive	1 (0.7)
Renal/urinary tracts	3 (2.3)
Allergy	0
Neurologic/psychiatric	22 (16.5)

CT, chemotherapy; B, bevacizumab.

### hs-CRP values and clinical outcome

We evaluated hs-CRP as a continuous variable, observing that increased hs-CRP levels were associated with decreased PFS (HR = 1.006, 95% CI 1.003–1.009, *p* < 0.0001) and OS (HR = 1.006, 95% CI 1.004–1.009, *p* < 0.0001). ROC curve analysis revealed that the best cutoff value of hs-CRP was 13.1 mg/L.

**Figure 2:**
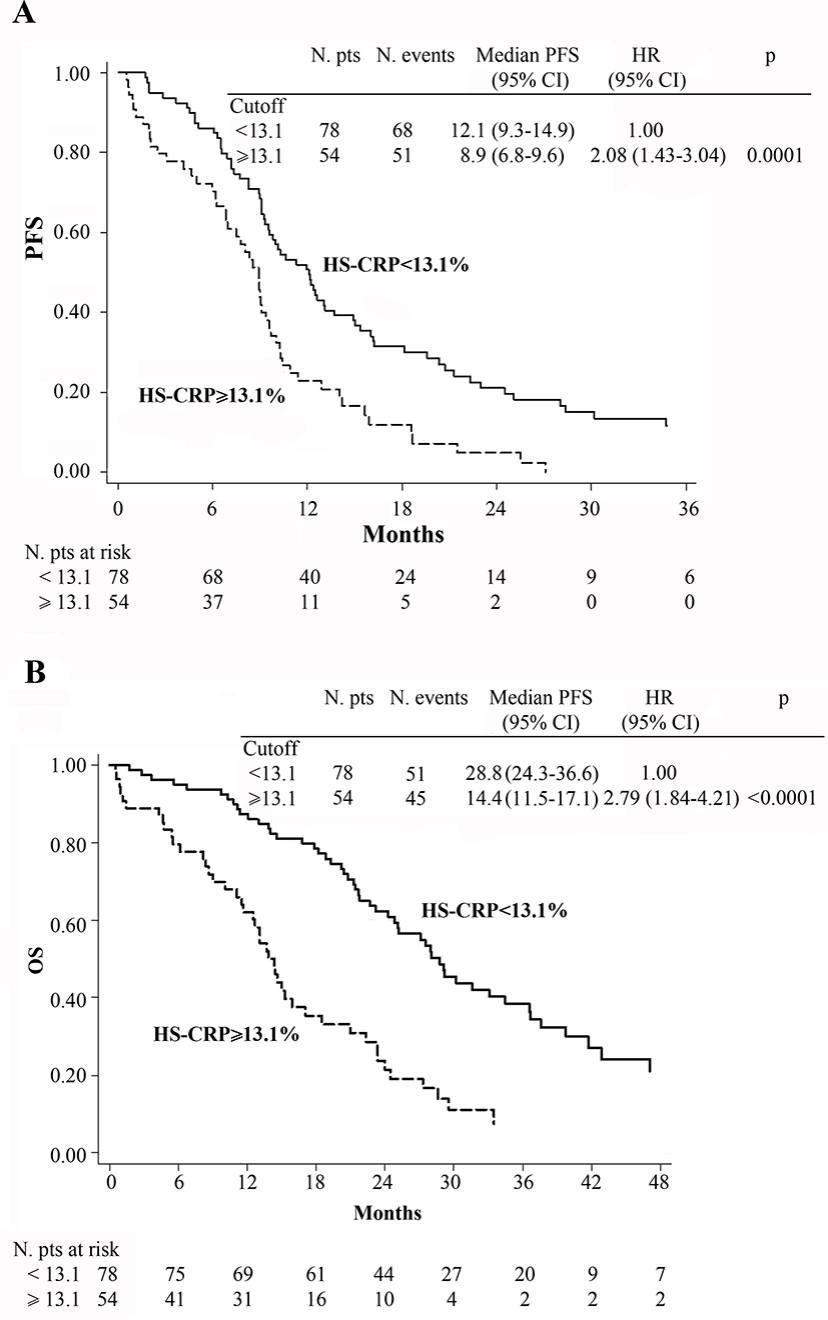
Kaplan-Meier curves of progression-free survival (PFS) (A), overall survival (OS) (B) for 132 patients

At baseline, patients with low hs-CRP levels (< 13.1 mg/L) had a median PFS of 12.1 months (95% CI 9.3–14.9) compared to 8.9 months (95% CI 6.8–9.6) for those with high hs-CRP levels (≥ 13.1 mg/L, HR = 2.08, 95% CI 1.43–3.04, *p* = 0.0001) (Figure 2A). Moreover, patients with low baseline hs-CRP had a median OS of 28.8 months with respect to 14.4 months for those with high baseline hs-CRP (HR = 2.79, 95% CI 1.84–4.21, *p* < 0.0001) (Figure 2B).

**Figure 3:**
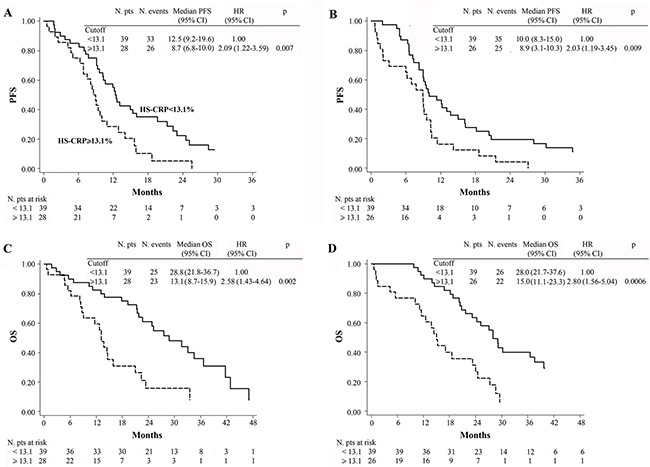
Kaplan-Meier curves of PFS in patients treated with chemotherapy plus bevacizumab (A), chemotherapy alone (B) and OS in patients treated with chemotherapy plus bevacizumab (C), chemotherapy alone (D)

Following stratification of patients in the 2 treatment arms (CT ± bevacizumab) according to baseline hs-CRP (< 13.1 mg/L vs. ≥ 13.1 mg/L), it was observed that PFS and OS were worse when hs-CRP was ≥ 13.1 mg/L, irrespectively of treatment (Figure 3A–3D). Interaction tests involving hs-CRP levels and treatment efficacy in the CT + B and CT-only groups were not statistically significant for either PFS (*p* = 0.577) or OS (*p* = 0.108).

We also collected hs-CRP values after 2 months of therapy in 107 patients, creating 4 categories according to baseline and second-sample hs-CRP levels: low-low, low-high, high-low and high-high. The study of dynamic hs-CRP levels showed that patients with low-low hs-CRP levels had the best median PFS (12.6 months, 95% CI 10.2–16.0) and OS (29.2 months, 95% CI 25.2–36.7) with respect to the other 3 categories (*p* = 0.002 and *p* = 0.0004 respectively) (Table 2).

**Table 2:** PFS and OS: stratification of 107 patients into different groups with respect to hs-CRP changes

		PFS					OS				
	No. patients	No. events	Median PFS (95% CI)	*p*	HR (95% CI)	*p*	No. events	Median OS (95% CI)	*p*	HR (95% CI)	*p*
**Low-Low**	58	48	12.6(10.2–16.0)		1.00		36	29.2(25.2–36.7)		1.00	
**Low-High**	9	9	7.4(2.9–10.0)		2.84(1.37–5.88)	0.005	7	18.2(3.7–33.1)		2.37(1.05–5.37)	0.038
**High-Low**	28	25	9.1(8.9–11.4)		1.95(1.18–3.21)	0.009	21	15.0(13.1–23.3)		2.54(1.47–4.39)	0.0008
**High-High**	12	12	9.4(3.1–15.7)	0.002	2.14(1.13–4.08)	0.020	10	14.8(9.0–28.6)	0.0004	3.02(1.48–6.20)	0.002

To evaluate hs-CRP modifications during the course of chemotherapy and to better understand the role of B in such changes, we considered PFS and OS after stratifying patients into 2 groups according to hs-CRP levels of the second blood sample. The first group included patients with low-low and high-low levels of hs-CRP, while the second included those with low-high and high-high hs-CRP. Patients in the former group had a median PFS of 11.4 months compared to 8.6 months for those in the latter group (HR 1.95, 95% CI 1.19–3.19, *p* = 0.008). Moreover, OS was 25.2 months in the first group and 15.3 months in the second group (HR 2.08, 95% CI 1.20–3.62, *p* = 0.009). However, this result was confirmed only in the CT-alone arm, where median PFS was 11.3 months in the first group and 7.4 months in the second group (HR 2.19, 95% CI 1.13–4.24, *p* = 0.02). There were no significant differences in PFS or OS between the 2 groups treated with CT plus B (Table 3).

**Table 3:** PFS and OS: stratification of 107 patients in two main groups with respect to hs-CRP changes after two months' therapy

		PFS					OS				
	No. patients	No. events	Median PFS (95% CI)	*p*	HR (95% CI)	*p*	No. events	Median OS (95% CI)	*p*	HR (95% CI)	*p*
**Overall**											
**Low – Low + High – Low**	86	73	11.4(9.3–14.0)		1.00		57	25.2(22.3–29.2)		1.00	
**Low – High + High –High**	21	21	8.6(6.1–10.3)	0.007	1.95(1.19–3.19)	0.008	17	15.3(11.5–28.6)	0.008	2.08(1.20–3.62)	0.009
**CT arm**											
**Low – Low + High – Low**	40	35	11.3(9.0–15.0)		1.00		26	27.3(23.3–36.6)		1.00	
**Low – High + High – High**	13	13	7.4(6.0–10.3)	0.017	2.19(1.13–4.24)	0.020	12	14.4(11.6–20.8)	0.0009	3.16(1.54–6.47)	0.002
**CT + B arm**											
**Low – Low + High – Low**	46	38	11.9(9.1–14.9)		1.00		31	24.8(19.3–34.5)		1.00	
**Low – High + High – High**	8	8	9.1(2.9–15.9)	0.204	1.64(0.76–3.54)	0.210	5	24.5(3.7-nr)	0.675	1.23(0.47–3.19)	0.675

nr:not reached.

**Figure 4:**
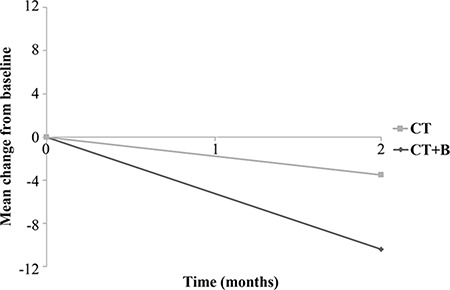
Mean change in hs-CRP from baseline in chemotherapy plus bevacizumab (CT + B) and chemotherapy (CT) arms

Interestingly, when we evaluated the mean change in hs-CRP values between the first and second samples, we observed a mean decrease of 10.4 mg/L from baseline in the CT plus B group (*p* = 0.029) and a mean decrease of 3.5 mg/L in the CT-alone group (*p* = 0.089) (Figure 4). We also considered drug toxicity as a single variable (grade 0–4) or a dichotomous variable (grade 0–2 vs. grade 3–4) but found no association between hs-CRP and toxicity.

### hs-CRP and VTE

**Figure 5:**
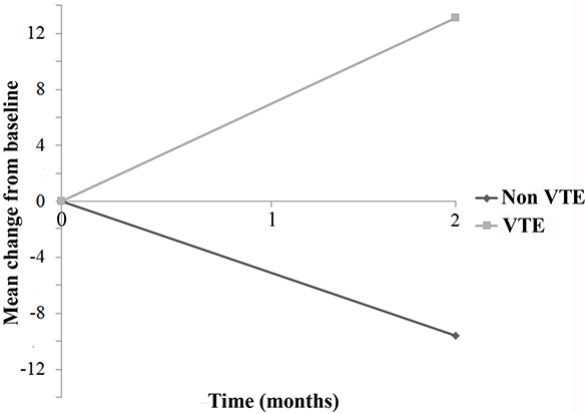
Mean change in hs-CRP from baseline as a function of venous thromboembolism (VTE)

The overall rate of thrombosis was 12.9% (17/132). The median baseline level of hs-CRP was 7.7 mg/L (range 0.7–301.0) in patients who did not experience VTE compared to 13.1 mg/L (range 1.8–301.0) in those who did (*p* = 0.174). The difference was greater, albeit not significantly, in the CT + B arm (6.6 mg/L in the no VTE group vs. 30.0 mg/L in the VTE group, *p* = 0.149). Evaluation of the mean change in hs-CRP values between the first and second samples revealed a mean increase of 13.1 mg/L in the thrombosis group (*p* = 0.020) and a mean decrease of 9.6 mg/L in those who did not experience a thrombotic event (*p* = 0.178) (Figure 5).

## DISCUSSION

Elevated levels of CRP have been described as a prognostic factor in various human malignancies, including tumors of the digestive system. The majority of studies assumed that elevated serum CRP levels in patients with malignancy constituted a bodily response, secondary to tumor necrosis, local tissue damage and inflammation caused by cytokines released from leukocytes infiltrating the tumor microenvironment, in particular IL-6 [23, 24]. Our study showed that high levels of hs-CRP at baseline were associated with poorer PFS and OS than low hs-CRP levels.

At present, only one paper has been published on the prognostic value of the hs-CRP in patients with colorectal cancer [25]. The retrospective study in question evaluated a population heterogeneous for clinical stage and chemotherapy. We assessed hs-CRP in patients with stage IV colorectal cancer at baseline and 2 months after beginning chemotherapy. As far as we know, our study is the first in the literature to assess the prognostic value of hs-CRP in patients with CRC treated with B. All the inflammatory conditions unrelated to the tumor that could have interfered with the hs-CRP assay were among the exclusion criteria of the ITACa trial [22].

Bevacizumab, a recombinant humanised monoclonal antibody directed against vascular endothelial growth factor (VEGF), was found to improve survival in both first- and second-line settings when added to chemotherapy. Notably, the results from the present study indicate that B is capable of cancelling the negative predictive value of high baseline hs-CRP (≥ 13.1 mg/L) that remain high after 2 months' therapy, but also of low baseline hs-CRP (< 13.1 mg/L) that increases during the same period of treatment.

One explanation for this could be that increased IL-6 levels (and, indirectly, hs-CRP values) are an indicator of elevated levels of VEGF [26, 27]. IL-6 enhances the production of VEGF by fibroblasts, thereby inducing angiogenesis [28]. Several studies have hypothesized that VEGF may stimulate angiogenesis in colon cancer, and this is strongly supported by results showing that bevacizumab-induced VEGF inhibition leads to decreased angiogenesis and the abrogation of cancer growth [22, 29].

Inflammation may interfere with various stages of hemostasis, either through the activation of coagulation or the inhibition of fibrinolysis and anticoagulant pathways [30]. As previously reported, the potential correlation between hs-CRP values and the risk of thrombosis and cardiovascular disease led us to evaluate the incidence of VTE in our patients. Although our data failed to confirm that hs-CRP is predictive of the risk of thromboembolic events, it is noteworthy that hs-CRP values in patients who developed thrombosis tended to increase during the course of treatment.

In conclusion, pre-treatment serum CRP levels may constitute a marker of aggressiveness in CRC patients. Elevated CRP levels prior to initial treatment would seem to be indicative of poor prognosis.

## PATIENTS AND METHODS

### The ITACa trial

Our study was conducted on 132 patients enrolled in the ITACa trial [22] for whom biological material was available. Patients were randomized to receive first-line chemotherapy (CT) (FOLFOX4 or FOLFIRI) only or CT plus bevacizumab (B). FOLFOX4 consisted of oxaliplatin 85 mg/m^2^ as a 2-hour infusion on day 1 and leucovorin 100 mg/m^2^ as a 2-hour infusion followed by bolus 5-FU 400 mg/m^2^ and a 22-hour infusion of 5-FU 600 mg/m^2^ on days 1–2 every 2 weeks. FOLFIRI consisted of the same 5-FU + leucovorin regimen with the addition of irinotecan 180 mg/m^2^ as a 90-minute infusion on day 1. B was administered as a 30- to 90-minute intravenous infusion at a dose of 5 mg/kg on day 1 of each 2-week cycle. Treatment was to be continued until disease progression (PD), withdrawal of consent or unacceptable toxicity, whichever came first. Tumor assessment tests were performed within 28 days of the start of the study treatment and repeated every 8 weeks during treatment until PD.

Response Evaluation Criteria in Solid Tumors (RECIST) guidelines were used to define all responses. An independent central review of patient scans was not carried out. Adverse events were graded according to the National Cancer Institute Common Toxicity Criteria (NCI-CTC) for Adverse Events, Version 3. We considered both deep vein thrombosis and pulmonary embolism for VTE. Thrombosis caused by infusion devices was excluded.

The study was performed in accordance with the principles of Good Clinical Practice and the ethical standards of the Declaration of Helsinki. The protocol was approved by the local Ethics Committee for each study site and written informed consent was obtained from each patient. Participation in this study was not mandatory for patients enrolled in the ITACa clinical trial.

### Blood collection

Samples of peripheral blood (3 ml) were collected from each patient at baseline and after 2 months' treatment with first-line chemotherapy. After collection, serum was separated by centrifugation and the supernatant was immediately transferred into cryovials and stored at −80°C.

### hs-CRP assay

After thawing serum samples at room temperature, hs-CRP was determined by particle enhanced immune-turbidometric assay using Cobas 6000 Analyser and dedicated reagents (Roche, Mannheim, Germany). Anti-CRP antibodies coupled to latex microparticles reacted with CRP in the sample to form an antigen/antibody complex. This led to agglutination and turbidity of the reaction mixture, which was proportional to the CRP concentration and measured quantitatively. The lower detection limit of the hs-CRP assay was 0.15 mg/L and the functional sensitivity 0.3 mg/L; the within-run precision (CV) was 1.6% at 0.54 mg/L and 0.4% at 15.9 mg/L, while the intermediate precision was 8.4% at 0.53 mg/L and 2.1% at 13.3 mg/L.

### Statistical analysis

The objectives of this secondary analysis were to examine the association between baseline hs-PCR levels and PFS and OS in the ITACa population, and to investigate their effect on PFS and OS of the addition of B to standard CT in the 2 different hs-PCR subgroups. The data cut-off for analysis was 31st December 2013, when the median duration of follow-up was 36 months (range 1–65).

PFS was defined as the time from random assignment to the first documentation of PD (as per investigator assessment), or death from any cause. Patients undergoing curative metastasectomy were censored at the time of surgery. OS was defined as the time interval between random assignment and death or last follow-up visit. PFS, OS and their two-sided 95% confidence intervals (95% CI) were estimated by the Kaplan-Meier method and curves were compared by the log-rank test (at a significance level of 5%).

The distribution of raw serum baseline hs-CRP data was skewed (data not shown) but was symmetric after log transformation. For this reason, the log-transformed hs-CRP level was used to observe the relationship between continuous hs-CRP levels and PFS and OS.

We performed ROC curve analysis to determine the best threshold of hs-PCR levels to predict PD at 9 months. Estimated hazard ratios (HRs) and their two-sided 95% CI were calculated using the Cox proportional-hazard model. HRs adjusted by baseline characteristics (gender, age, performance status, *KRAS* status, tumor localization (rectum/colon) and chemotherapy regimen (FOLFOX4/FOLFIRI)) were calculated using the Cox proportional-hazard model. Covariate selection was based on a list of suspected prognostic factors derived from the ITACa study [22].

The effect of the interaction between hs-CRP levels and treatment on PFS/OS was evaluated using Cox regression models of the entire population (CT + B and CT only arms) including hs-CRP levels, treatment, and treatment by hs-CRP levels. We also used nonparametric test to examine the potential correlation between baseline/second sample hs-CRP levels and disease progression or therapy regimen in 107 patients for whom sequential hs-CRP data were available. All *p* values were based on two-sided testing. Statistical analyses were performed using SAS statistical software version 9.3 (SAS Inc., Cary, NC, USA).
